# TICAM2-related pathway mediates neutrophil exhaustion

**DOI:** 10.1038/s41598-020-71379-y

**Published:** 2020-09-01

**Authors:** RuiCi Lin, Yao Zhang, Kisha Pradhan, Liwu Li

**Affiliations:** 1 Translational Biology, Medicine, and Health Graduate Program, Virginia Tech, Blacksburg, VA 24061 USA; 2 Department of Biological Sciences, Virginia Tech, Blacksburg, VA 24061 USA

**Keywords:** Immunology, Inflammation, Innate immune cells, Innate immunity

## Abstract

Pathogenic inflammation and immune suppression are the cardinal features that underlie the pathogenesis of severe systemic inflammatory syndrome and sepsis. Neutrophil exhaustion may play a key role during the establishment of pathogenic inflammation and immune suppression through elevated expression of inflammatory adhesion molecules such as ICAM1 and CD11b as well as immune-suppressors such as PD-L1. However, the mechanism of neutrophil exhaustion is not well understood. We demonstrated that murine primary neutrophils cultured in vitro with the prolonged lipopolysaccharides (LPS) stimulation can effectively develop an exhaustive phenotype resembling human septic neutrophils with elevated expression of ICAM1, CD11b, PD-L1 as well as enhanced swarming and aggregation. Mechanistically, we observed that TICAM2 is involved in the generation of neutrophil exhaustion, as TICAM2 deficient neutrophils have the decreased expression of ICAM1, CD11b, PD-L1, and the reduced aggregation following the prolonged LPS challenge as compared to wild type (WT) neutrophils. LPS drives neutrophil exhaustion through TICAM2 mediated activation of Src family kinases (SFK) and STAT1, as the application of SFK inhibitor Dasatinib blocks neutrophil exhaustion triggered by the prolonged LPS challenge. Functionally, TICAM2 deficient mice were protected from developing severe systemic inflammation and multi-organ injury following the chemical-induced mucosal damage. Together, our data defined a key role of TICAM2 in facilitating neutrophil exhaustion and that targeting TICAM2 may be a potential approach to treating the severe systemic inflammation.

## Introduction

Sepsis, a systemic inflammatory reaction to severe infection/injury leading to multi-organ failure, remains one of the leading causes of death for hospitalized patients with no effective cure^1^. The fundamental feature of sepsis is characterized by the dysregulated inflammatory response, exemplified by an early phase of “cytokine storm” followed by a late phase of pathogenic inflammation and immune suppression, which collectively contribute to multi-organ failure due to excessive tissue damage and secondary infection^2,3^. However, cellular and molecular mechanisms that are responsible for the pathogenic inflammation and immune suppression are not well understood.

Neutrophils are the most abundant leukocyte in circulation and play an essential role in sepsis as the first line of defense against microbial invasion. Equipped with an arsenal of antimicrobial proteins, neutrophils exert both intracellular and extracellular microbicidal abilities initiating the pro-inflammatory reaction during sepsis^4^; and the interaction between neutrophils and other immune cells is required for the resolution of excessive inflammation as well as effective host defense^5^. However, “exhausted” neutrophils from sepsis patients often exhibit pathogenic and immune-suppression phenotype characterized by the elevated expression of immunosuppression-associated markers (i.e. PD-L1) as well as adhesion molecules (i.e. CD11b, CD29, and ICAM1), which control neutrophil recruitment to the site of inflammation and extravasation, leading to their pathogenic aggregation within vital tissues^6–12^. Indeed, studies suggest that the degree of neutrophil dysfunction or exhaustion as well as their accumulation within vital organs are directly correlated with the severity of the sepsis outcome^13–15^. Despite its clinical significance, underlying mechanisms of neutrophil exhaustion leading to the pathogenic inflammation during sepsis are still poorly studied.

Toll-like receptor 4 (TLR4) is one of the pattern-recognition receptors (PRR) that can be activated by pathogen-associated molecular patterns (PAMPs), such as bacterial endotoxin^16^. LPS-induced TLR4 signaling modulates neutrophil activation during the pathogenesis of sepsis. Two divergent pathways downstream of TLR4 include MyD88-dependent and TICAM2 (TRAM)/TRIF-dependent (MyD88-independent) signaling networks. The MyD88-dependent pathway activates nuclear factor κB (NF-κB) in a mitogen-activated-protein-kinases-dependent manner, and the TICAM2/TRIF-dependent pathway triggers interferon regulator factor 3 (IRF-3)-mediated responses, resulting in the late activation of NF-κB^17^. The MyD88- and TICAM2/TRIF-dependent pathways are complex and context-dependent, with downstream effector processes still not fully defined^18,19^. In the context of sepsis, the effects of the MyD88 signaling pathway are inconclusive as the presence of MyD88 has been reported to be beneficial or detrimental for sepsis outcomes^20–24^, on the other hand, the role of the TICAM2/TRIF-dependent pathway in neutrophil exhaustion during the sepsis pathogenesis is not well understood.

Although functional and phenotypic changes relevant to neutrophil exhaustion from human septic patients have been identified, the lack of an appropriate in vitro system to capture neutrophil exhaustion in vitro hinders the study of underlying mechanisms. In the present study, we examined the role of TICAM2 (TRAM), which is an adaptor protein necessary for the recruitment of TRIF in the MyD88-independent pathway, in neutrophil exhaustion related to sepsis. We stimulated C57BL/6 WT and TICAM2 knockout (KO) murine bone marrow (BM) derived neutrophils with prolonged LPS treatment to recapitulate neutrophil exhaustion. Additionally, we utilized the DSS-induced murine acute mucosal damage model, a well-controlled model mimicking human ulcerative injury^25,26^, with severe cases leading to systemic multi-organ injury and sepsis, to examine neutrophil exhaustion in vivo. Our data demonstrate that with the prolonged LPS challenge, neutrophils can be exhausted in vitro with the cardinal characteristics of enhanced expression of ICAM1, CD11b, CD29, PD-L1, reduced expression of CD62L and CXCR2, as well as enhanced swarming/aggregation. We found that neutrophil exhaustion is attenuated in TICAM2 deficient neutrophils. We further observed that SFK-mediated STAT1 activation under the control of TICAM2 is responsible for neutrophil exhaustion. In vivo, we showed that TICAM2 deficient mice are protected from systemic inflammation with improved survival, lower clinical scores, and less neutrophil exhaustion. Together, our results shed light on the significance of the less defined TICAM2 (TRAM)-pathway in neutrophil exhaustion related to systemic inflammation and sepsis pathogenesis.

## Results

### LPS-mediated neutrophil exhaustion is partially dependent upon TICAM2

**Figure 1:**
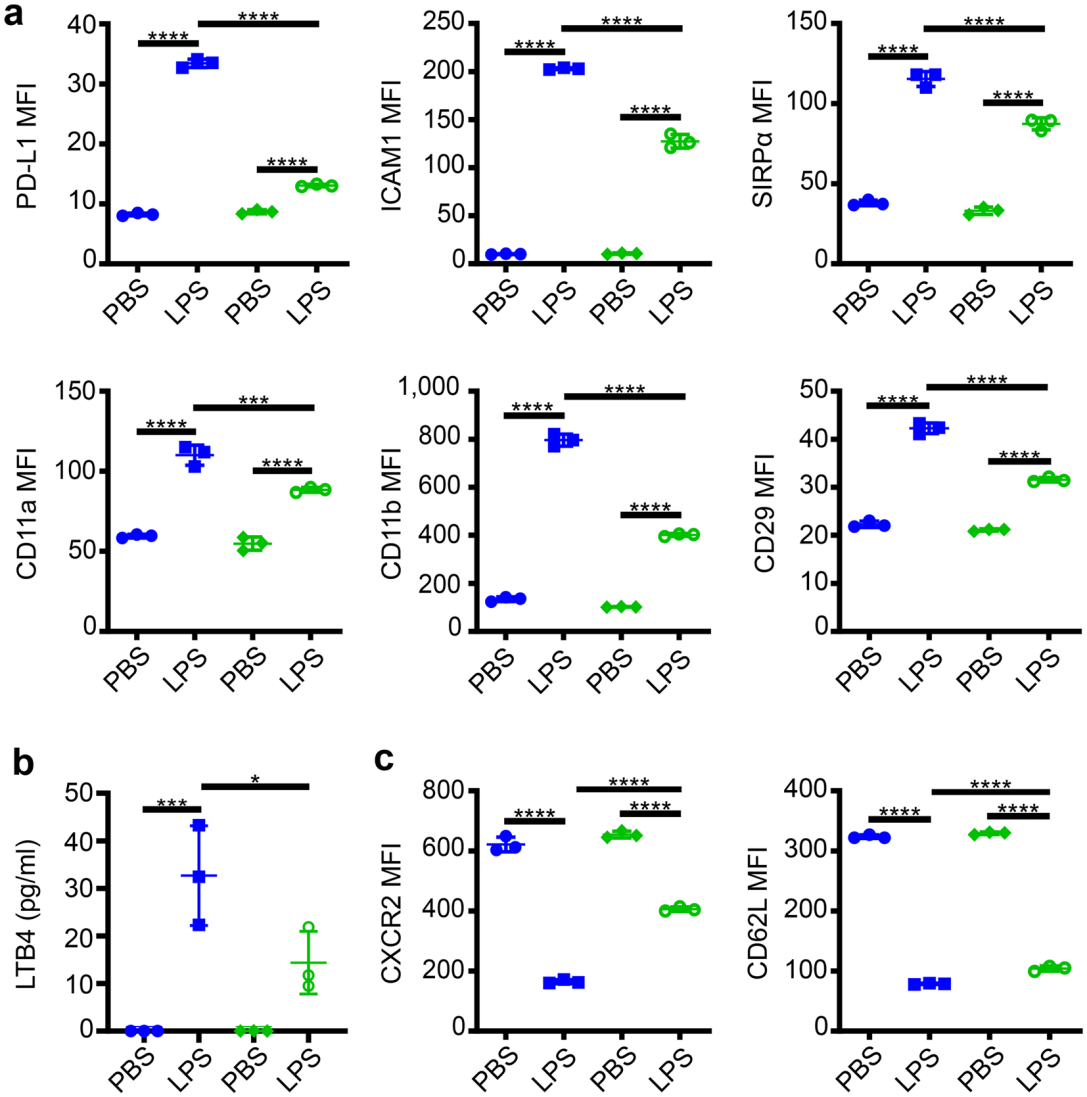
Influence of prolonged LPS stimulation on WT and TICAM2 KO neutrophil cell surface markers and secreted chemokine profile. (**a**) The expression of PD-L1, ICAM1, SIRPα, CD11a, CD11b, and CD29 on WT (blue) and TICAM2 KO (green) neutrophils treated with PBS or LPS (100 ng/ml) for 24 h (n = 3). (**b**) The release of LTB4 from WT and TICAM2 KO neutrophils treated with PBS or LPS (100 ng/ml) for 24 h (n = 3). (**c**) The levels of CXCR2 and CD62L on WT and TICAM2 KO neutrophils treated with PBS or LPS (100 ng/ml) for 24 h (n = 3). Blue: WT, Green: TICAM2 KO. All n-numbers represent data derived from individual cell cultures with data plotted as mean ± SD. The absolute values that are below the minimum detectable dose (3.7 pg/ml) are represented with 0 pg/ml (**c**). ****P < 0.0001, ***P < 0.001, *P < 0.05 using one-way ANOVA test followed by the post-hoc Sidak multiple comparisons test.

First, we tested key markers of neutrophil exhaustion with in vitro cultured murine BM neutrophils with prolonged LPS challenge. To mimic murine septic condition, 100 ng/ml LPS was chosen for further study^27,28^. Neutrophils treated with LPS for 24 h were characterized with significantly increased expression of PD-L1, ICAM1, SIRPα, CD11a, CD11b, CD29 (Fig. 1a), and LTB4 (Fig. 1b) as well as significantly reduced expression of CD62L and CXCR2 (Fig. 1c). Together, our data reveal that murine neutrophils with prolonged LPS stimulation recapitulate key features of neutrophils from sepsis patients and those with high risks of organ failure, including upregulated PD-L1^9^, ICAM1^6,7^, CD29^8^ and downregulated CD62L^29,30^ as well as CXCR2^10,31^.

Next, we tested the potential role of less-characterized TICAM2 during neutrophil exhaustion in vitro. We stimulated neutrophils collected from TICAM2 KO murine bone marrow with 100 ng/ml LPS for 24 h as mentioned above to induce neutrophil exhaustion. As compared to WT neutrophils, the induction levels of PD-L1, ICAM-1, SIRPα, CD11a, CD11b, CD29, and LTB4 by LPS were significantly attenuated in TICAM2 KO neutrophils as compared to WT neutrophils (Fig. 1a,b). On the other hand, the suppression magnitudes of CXCR2 and CD62L by LPS in TICAM2 KO neutrophils were partially abolished as compared to WT neutrophils (Fig. 1c). Our data reveal that TICAM2 KO neutrophils exhibit reduced exhaustion characteristics as compared to WT neutrophils in response to prolonged LPS stimulation, suggesting that TICAM2 is partially required for inducing the maximum neutrophil exhaustion.

### TICAM2 is responsible for elevated swarming of exhausted neutrophils in vitro

**Figure 2:**
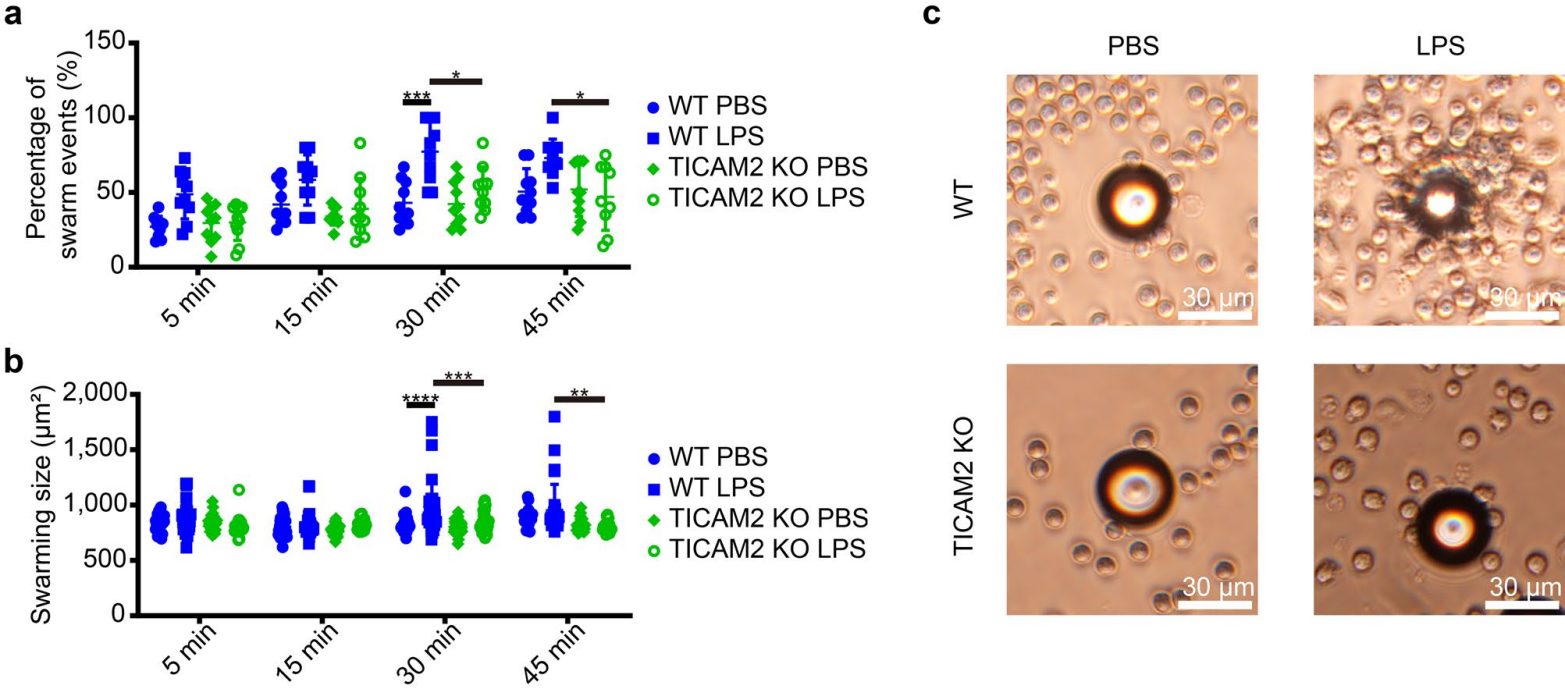
TICAM2-mediated changes to the neutrophil swarming patterns in response to LPS stimulation. (**a**,**b**) The percentage of swarm events (**a**) and the swarming size (**b**) of PBS- or LPS-stimulated (100 ng/ml; 24 h) WT and TICAM2 KO neutrophils co-incubated with polystyrene beads (diameter = 30 µm) for 5, 15, 30, and 45 min (n = 10). (**c**) The representative images of swarms from each group shown in (**a**,**b**) at the 30-min time point. Blue: WT, Green: TICAM2 KO. All n-numbers represent data derived from separate fields under the light microscope with ×400 magnification, and the data are plotted as mean ± SD. Neutrophil to bead ratio in co-incubation was 40:1. Image scale bars = 30 µm. ****P < 0.0001, ***P < 0.001, **P < 0.01, *P < 0.05 using the two-way ANOVA test followed by the post-hoc Tukey’s multiple comparisons test (**a**,**b**).

Exhausted neutrophils from septic patients with elevated levels of adhesion molecules including CD11b, ICAM1, and CD29 as well as lipid mediator LTB4 tend to exhibit an aggravated swarming phenotype within vital tissues, further exacerbating tissue damage^1,32^. Thus, we compared the swarming behaviors of WT and TICAM KO neutrophils with prolonged LPS challenge in vitro. After stimulation with PBS or 100 ng/ml LPS for 24 h, neutrophils were further incubated together with 30 um-diameter polystyrene beads at a ratio of 40:1 (neutrophil to bead) and allowed to swarm for 5, 15, 30, and 45 min. We then measured the swarming size (the size of aggregation formed by neutrophils attaching to the bead) and the percentage of swarming events (beads that were attached by neutrophils per field/total beads per field) as indicators of the neutrophil swarming behavior. The average percentage of WT neutrophil swarming events in the LPS group exceeded 50% within 5-min incubation, while it was around 25% in the PBS group (Fig. 2a). In both groups, the swarming size and the number of swarming events elevated through the time course (Fig. 2a,b). However, by the 30-min time point, not only was the swarming sizes but also the percentage of swarming events was ~ 1.5 times greater in WT neutrophils challenged with LPS as compared to WT neutrophils treated with PBS control, confirming that exhausted neutrophils exhibit an elevated swarming phenotype (Fig. 2a,b). Prior studies have shown the decreased levels of chemotaxis^33^ and phagocytosis^34^ as well as the elevation of swarming/adhesiveness^35^ in neutrophils from septic subjects, which are correlated with the severity of sepsis^36^. Moreover, increased sizes of neutrophil swarming were observed in patients after sepsis^37^, which is in concert with our results, indicating that the phenotypic characteristics of exhausted neutrophils with elevated swarming can be captured in vitro with cultured murine primary neutrophils.

In contrast, TICAM2 KO neutrophils challenged with either PBS or LPS exhibited similar swarming ability with no statistical difference in terms of the swarming size or the percentage of swarming events through the entire incubation period (Fig. 2a,b). Compared to WT exhausted neutrophils, both the swarming sizes and percentages of swarming events were significantly lower in TICAM2 KO exhausted neutrophils at 30- and 45-min time points of the co-incubation period (Fig. 2a–c). Together, our data suggest that TICAM2 is required for not only the expression of surface markers of neutrophil exhaustion but also for the related swarming phenotype associated with neutrophil exhaustion.

### TICAM2 mediates STAT1 phosphorylation and SFK induction in exhausted neutrophils in vitro

**Figure 3:**
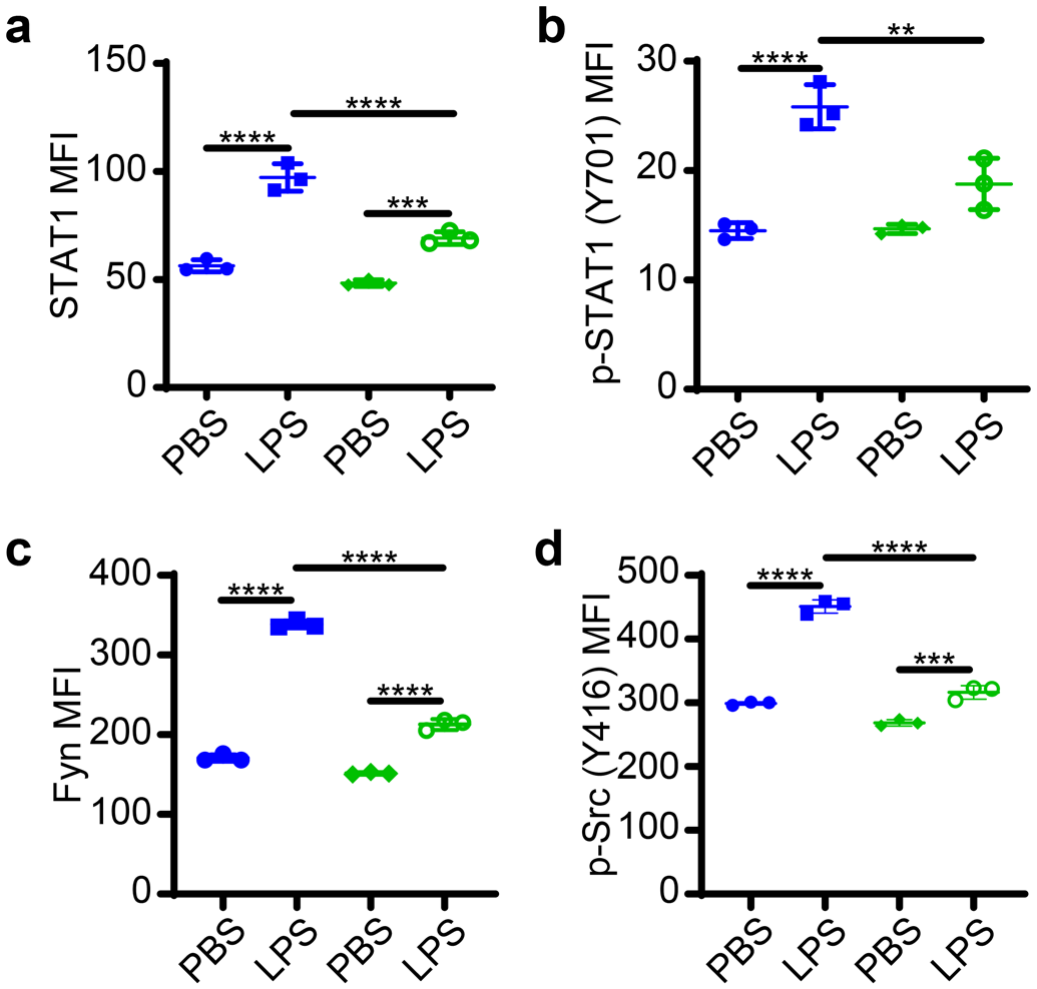
TICAM2-mediated STAT1 and Src kinases expression and activation in exhausted neutrophils. (**a**) The total STAT1 level on WT and TICAM2 KO neutrophils treated with PBS or LPS (100 ng/ml) for 24 h (n = 3). (**b**) The expression of phosphorylated STAT1 at Tyr701 on WT and TICAM2 KO neutrophils treated with PBS or LPS (100 ng/ml) for 24 h (n = 3). (**c**) Fyn expression on WT and TICAM2 KO neutrophils stimulated with PBS or LPS (100 ng/ml) for 24 h (n = 3). (**d**) The level of phosphorylated Src at Tyr416 on WT and TICAM2 KO neutrophils stimulated with PBS or LPS (100 ng/ml) for 24 h (n = 3). Blue: WT, Green: TICAM2 KO. All n-numbers represent data derived from individual cell cultures with data plotted as mean ± SD unless otherwise stated. ****P < 0.0001, ***P < 0.001, **P < 0.01 using the one-way ANOVA test followed by the post-hoc Sidak multiple comparisons test.

TICAM2 mediated pathway was known to activate IRF3 and STAT1^38^. The process of tyrosine phosphorylation has been implicated during the activation of STAT1^39^. With the particular interest, the Src family kinase (SFK), a family of non-receptor tyrosine kinases, is a known component of the TLR signaling network^40^. Johnsen et al*.* reported that Src kinase is necessary for the activation of IRF3 and STAT1 mediated by TLR3^41^. Furthermore, the expression and activation of STAT1 have been shown to be indispensable for promoting inflammation^42^ and regulating PD-L1^43^ as well as ICAM1 expression^44^, which are signatures of exhausted neutrophils. Based on these clues, we tested whether TICAM2 is responsible for LPS-mediated activation of STAT1 in exhausted neutrophils. We examined the levels of STAT1 and phosphorylated STAT1 through the well-established and quantifiable assay of flow cytometry with previously reported and verified antibodies^45,46^. We found that the LPS challenge significantly increased the total levels of STAT1 by ~ 100% as well as the phosphorylated STAT1 (p-Tyr 701) levels by ~ 90% in WT neutrophils (Fig. 3a,b). In contrast, the induction magnitude of STAT1 as well as p-STAT1 by LPS in TICAM2 KO neutrophils were significantly lower as compared to WT neutrophils (Fig. 3a,b), which is in line with the prior report collected in macrophages indicating that TICAM2-TRIF pathway is required for STAT1 activation in response to LPS^47^. Src tyrosine family kinases (SFK) such as Fyn and Src capable of activating STAT1 were shown to be activated downstream of TLR4 in other cellular systems^48^. Thus, we tested the status of Fyn and Src in WT and TICAM2 KO neutrophils exhausted by prolonged LPS challenge, by staining with specific antibodies followed by flow analysis and quantification^49^. We found that the level of Fyn was significantly induced in WT LPS-stimulated exhausted neutrophils as compared to PBS-treated neutrophils, while the LPS-induced Fyn level in TICAM2 KO neutrophils was dramatically lower than their WT counterparts (Fig. 3c). Although the relative induction is less pronounced, LPS also induced Src activation in WT neutrophils but less in TICAM2 KO neutrophils (Fig. 3d).

### Suppression of SFK mitigates neutrophil exhaustion in vitro

**Figure 4:**
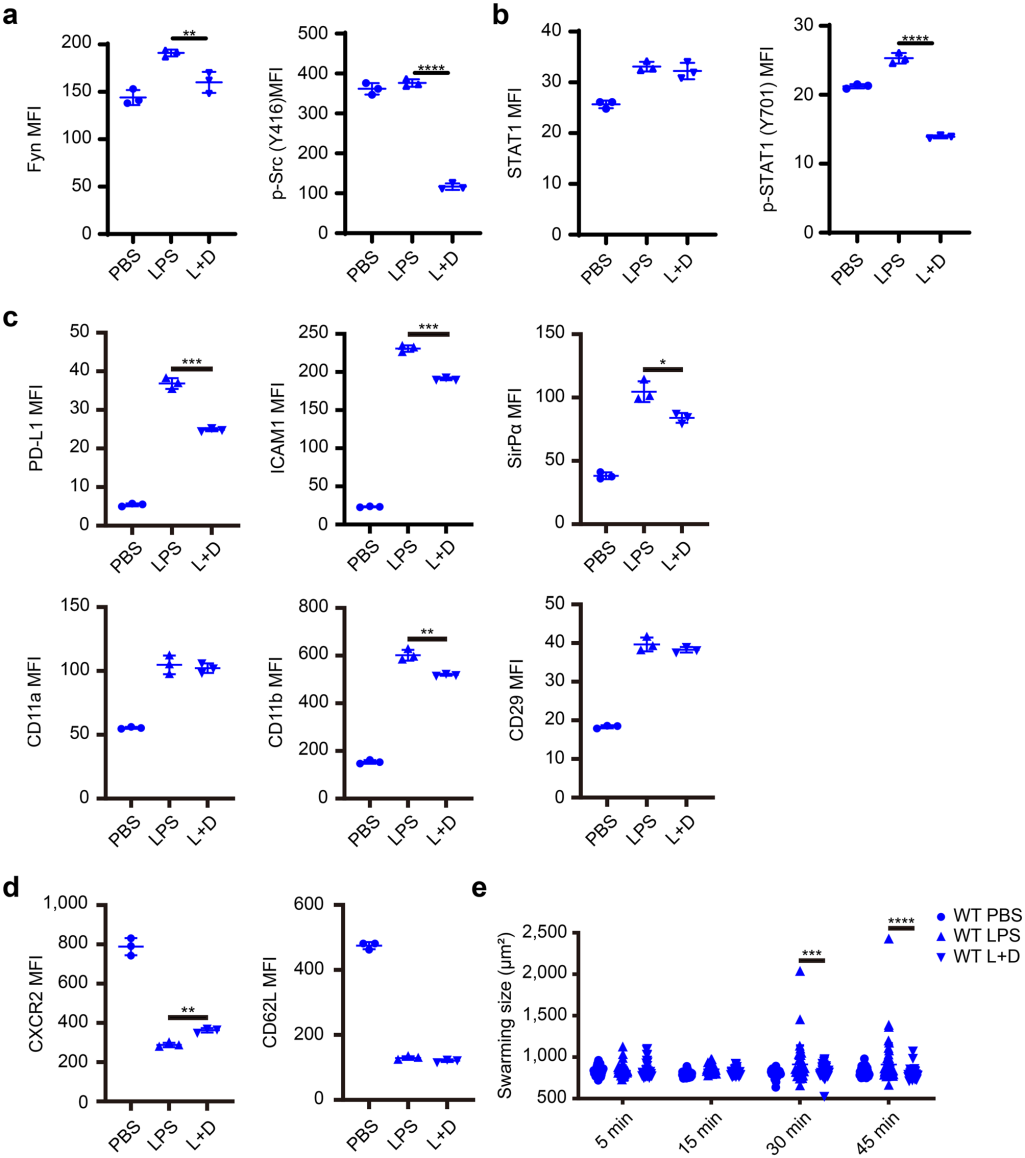
SFK-mediated phenotypical and functional neutrophil exhaustion. (**a**) The expression of Fyn and phosphorylated Src at Tyr416 on WT neutrophils stimulated with PBS or LPS (100 ng/ml) in the presence of Dasatinib (100 nM) for 24 h (n = 3). (**b**) The total STAT1 and activated STAT1 at Tyr701 on WT neutrophils treated with PBS or LPS (100 ng/ml) in the presence of Dasatinib (100 nM) for 24 h (n = 3). (**c**,**d**) The levels of PD-L1, ICAM1, SIRPα, CD11a, CD11b, CD29 (**c**), CXCR2, and CD62L (**d**) on WT neutrophils treated with PBS or LPS (100 ng/ml) in the presence of Dasatinib (100 nM) for 24 h (n = 3). (**e**) The swarming size of PBS- or LPS-stimulated (100 ng/ml; 24 h, with or without 100 nM Dasatinib) WT neutrophils co-incubated with polystyrene beads (diameter = 30 µm) for 5, 15, 30, and 45 min (n = 10; each data point is derived from separate fields under the light microscope with  ×400 magnification). All n-numbers represent data derived from individual cell cultures with data plotted as mean ± SD unless otherwise stated. Neutrophil to bead ratio in co-incubation was 40:1. ****P < 0.0001, ***P < 0.001, **P < 0.01, *P < 0.05 using Student’s t-test (**a**–**e**). L, LPS; D, Dasatinib.

Afterward, we tested whether the Src family kinases (SFK) may be required for STAT1 activation in neutrophils by applying Dasatinib, the selective SFK inhibitor. Indeed, Dasatinib application dose-dependently inhibited the phosphorylation of Src, with 100 nM Dasatinib yielding the most robust inhibitory effect in blocking Fyn and p-Src expression (Supplementary Fig. 1). In the presence of Dasatinib, the elevation of Fyn induced by LPS was abolished in WT neutrophils. Although Src phosphorylation was less pronounced by LPS, cells co-treated with Dasatinib and LPS exhibited significantly lower levels of phosphorylated Src as compared to PBS control neutrophils (Fig. 4a). In spite of no change in the total STAT1 level, the level of activated p-STAT1 was significantly reduced in LPS-exhausted neutrophils co-incubated with Dasatinib (Fig. 4b), suggesting that the STAT1 phosphorylation instead of the total STAT1 requires the activation of SFK.

Further, in order to determine if SFK plays a causal role in inducing neutrophil exhaustion, we tested the efficacy of blocking SFK with selective inhibitor Dasatinib during neutrophil exhaustion by LPS. We treated neutrophils challenged with PBS or LPS in the presence of 100 nM Dasatinib. The levels of PD-L1, ICAM1, SIRPα, and CD11b, but not CD11a and CD29, were significantly attenuated in exhausted neutrophils co-incubated with Dasatinib, although still greater than in control neutrophils, as compared to LPS treated neutrophils (Fig. 4c). In addition, the expression of CXCR2 but not CD62L was restored in LPS-exhausted neutrophils co-incubated with Dasatinib (Fig. 4d).

We then tested whether the blockage of SFK may not only block the alterations of surface markers representative of neutrophil exhaustion but also ameliorate functional exhaustion as represented by altered swarming phenotype. We observed that the average swarming size of LPS-exhausted neutrophils co-treated with Dasatinib was significantly reduced after 30-min co-incubation, reaching to only ~ 75% swarming size of LPS-exhausted neutrophil without the addition of Dasatinib (Fig. 4e). Taken together, our data suggest that LPS may cause neutrophil exhaustion through SFK and that the application of SFK inhibitor may partially block the generation of neutrophil exhaustion at both phenotypic as well as functional levels.

### Neutrophil exhaustion requires TICAM2 in vivo

**Figure 5:**
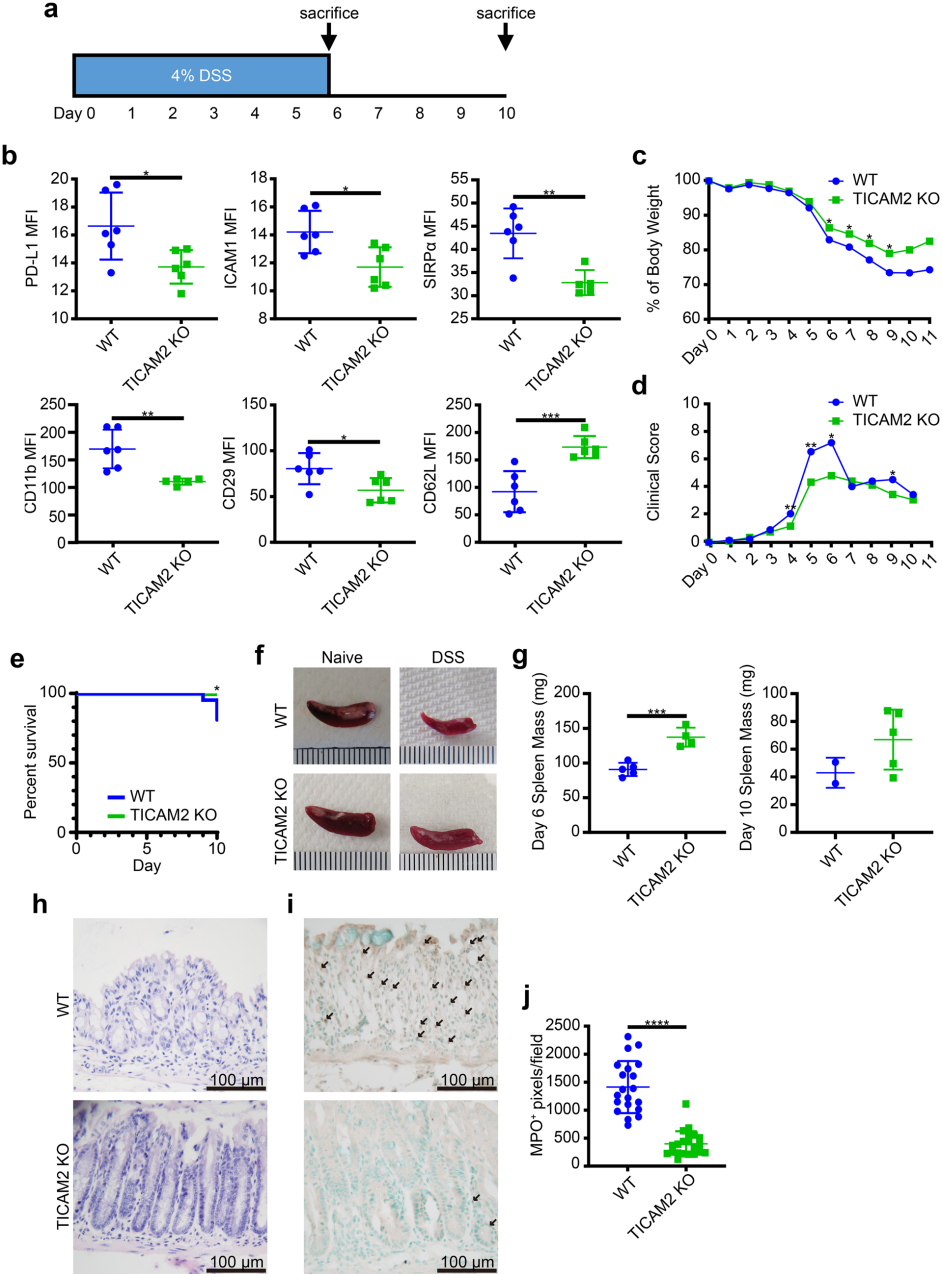
The ameliorated neutrophil exhaustion and mitigated symptoms in TICAM2 KO septic mice. (**a**) A schematic protocol of DSS treatment. (**b**) The expression of PD-L1, ICAM1, SIRPα, CD11b, CD29, and CD62L on spleen-resident neutrophils harvested at the time of sacrificing (day 10) from WT and TICAM2 KO DSS-induced septic mice (n = 5 or 6). (**c**,**d**) Changes of body weight (**c**) and clinical scores including stool consistency and bleeding (**d**) of WT and TICAM2 KO mice throughout the DSS treatment (n = 5 or 6). Values were expressed as means. (**e**) The Kaplan–Meier survival plot of WT and TICAM2 KO DSS-induced septic mice. (**f**) Representative images of spleens from WT septic mouse, WT naïve mouse, TICAM2 KO naïve mouse, and TICAM2 KO septic mouse (from quadrant 1 to 4) collected on day 10. (**g**) The spleen mass of WT and TICAM2 KO DSS-induced septic mice on day 6 (left) and day 10 (right) (n = 2, 4, or 5). (**h**) H&E-stained sections of colons from WT (upper) and TICAM2 KO mice (lower) with DSS treatment. (**i**) Immunohistological staining of MPO in colon tissue of WT and TICAM2 KO mice administrated with DSS treatment. Arrows indicate MPO^+^ neutrophils (brown). (**j**) Quantitative analysis of MPO staining in colons from WT and TICAM2 KO mice treated with DSS (n = 20; each data point is derived from separate fields under the light microscope with  ×400 magnification). All n-numbers represent data derived from separate mice with data plotted as mean ± SD unless otherwise stated. ****P < 0.0001, ***P < 0.001, **P < 0.01, *P < 0.05 using Student’s t-test (**b**–**d**,**g**,**j**) and Log-rank test (**e**). Image scale bars = 100 um. Data are representative of three independent experiments. MPO, myeloperoxidase.

Finally, we tested the in vivo role of TICAM2 during neutrophil exhaustion. WT and TICAM2 KO mice were fed with 4% DSS water for 6 consecutive days to chemically induce acute and severe gut damage, leading to systemic inflammation and multi-organ injury reminiscent of sepsis (Fig. 5a). Consistent with our in vitro data of neutrophil exhaustion documented above, we observed that circulating neutrophils collected from WT mice on day 6 post DSS challenge expressed significantly less CD62L and CXCR2 than those from WT naïve groups (Supplementary Fig. 2a). On the other hand, higher levels of PD-L1, ICAM1, SIRPα, and CD29 but lower levels of CD62L as well as CXCR2 were noticed on spleen-resident neutrophils from DSS-challenged mice as compared to their naïve counterparts (Supplementary Fig. 2b), demonstrating that neutrophil exhaustion can be observed in the murine mucosal-injury model in vivo. Although there was no statistical difference between the levels of exhaustion-related surface markers on circulating blood neutrophils from DSS-treated WT and TICAM2 KO mice (Supplementary Fig. 2c), PD-L1, ICAM1, SIRPα, CD11b, and CD29 levels were significantly reduced while the level of CD62L was significantly elevated on spleen-resident neutrophils from DSS-treated TICAM2 KO mice as compared to DSS-treated WT mice on day 10 post DSS challenge (Fig. 5b), suggesting that TICAM2 is required for inducing the full extent of neutrophil exhaustion not only in vitro but also in vivo.

Since neutrophil exhaustion has been correlated with elevated sepsis mortality^50^, we tested the hypothesis that TICAM2 KO mice with DSS-triggered acute gut damage may exhibit less-severe systemic injury and mortality as compared to WT counterparts. Indeed, from day 6 to day 9, WT mice suffered from significantly severe weight loss as compared to TICAM2 KO mice (Fig. 5c). In addition, TICAM2 KO septic mice had significantly lower clinical scores, including intestinal bleeding and stool consistency, as compared to WT mice from day 4 to day 6 (Fig. 5d). Overall, TICAM2 KO mice exhibited significantly higher survival (100%) by the end of the 10-day observation period as compared to WT mice (80%) (Fig. 5e).

The shrinkage of spleen size was observed in DSS-treated mice (Fig. 5f), and the mass (mg) of spleen from DSS-treated WT mice was only ~ 65% of that from DSS-treated TICAM2 KO mice on day 6 and day 10 (Fig. 5g). Furthermore, the histological analysis revealed that, compared to TICAM2 KO septic mice, DSS-treated WT mice exhibited widespread inflammation throughout the mucosa characterized by the shrinkage of villi, loss of crypts (Fig. 5h), and enhanced neutrophils infiltration (Fig. 5j,i). Collectively, our data reveal that TICAM2 KO mice exhibit attenuated neutrophil exhaustion with significantly improved survival outcomes when challenged with DSS as compared to WT mice.

## Discussion

Despite the advancement of critical care and supportive therapies^51^, the incidence of sepsis remains high, with ~ 30 million people worldwide being affected and ~ 6 million deaths annually^52^, due to the lack of effective cure. The progression of sepsis is correlated with the dysregulation of innate immune cells including neutrophils, with neutrophil dysfunction and/or exhaustion leading to elevated mortality and morbidity post sepsis^53^. However, the mechanism of neutrophil exhaustion in sepsis is not well understood. Our present study reveals that TICAM2 contributes to the development of exhausted neutrophils through activating SFK. We found that TICAM2 deficiency lowered the induction of PD-L1, ICAM1, SIRPα, CD11a, CD11b, CD29, and LTB4, and attenuated the reduction of CXCR2 and CD62L on LPS-induced exhausted neutrophils. At the functional level, we document that TICAM2-deficient neutrophils exhibit attenuated exhaustion, as reflected on the reduction of dysregulated swarming in vitro. At the translational level, deficiency in TICAM2 provides protective effects on DSS-induced systemic inflammation, reduced tissue injury, and improved survival.

Our findings complement and extend previous reports about the crucial roles of exhausted neutrophils in sepsis. Neutrophils collected from septic patients are known to have the altered expression of key surface regulatory molecules involved in migration, adhesion, transmigration, antimicrobial activity, and lifespan^1^. The clinical features of septic mouse models, in which neutrophils displayed abnormal functions, bear the resemblance to those of human septic patients^54,55^. In agreement with these reports, our data show that sepsis-related neutrophil exhaustion indeed can be recapitulated by in vitro prolonged LPS stimulation, manifested by abnormal swarming behaviors. We then examined key adhesion/aggregation-related markers, including ICAM1^56,57^, CD11a, CD11b^58^, and CD29^59^ associated with aggravated neutrophil swarming in vital organs. By using flow cytometry analysis which enables quantitative measurement of target proteins within the limited amount of neutrophils^7,8,60^, we observed that the levels of these key markers were significantly increased on WT exhausted neutrophils, which is in line with previous reports using neutrophils from human septic patients^61,62^. Of note, neutrophils expressing high ICAM1 have been reported to undergo reverse transendothelial migration (rTEM) and generate excess ROS which collectively contribute to the systemic inflammatory responses leading to excessive tissue damage^63^. Furthermore, we examined other key markers associated with the immunosuppressive effects of exhausted neutrophils. The levels of immunosuppressive molecule PD-L1 and phagocytosis inhibitory molecule SIRPα are significantly elevated on WT exhausted neutrophils, which correlate closely with the immunosuppression phenotype favoring the augmentation of Th2 cells and depression of Th1 cells as well as the reduced neutrophil phagocytic potential to combat bacterial infection during the late stage of sepsis^21,64,65^. On the contrary, the expression of CXCR2 and CD62L drops significantly, a phenomenon that is shown in aged neutrophils as well as neutrophils from septic patients^10,29–31,66,67^. Together, our current data re-affirm the value of using murine primary neutrophils in studying the fundamental principles of neutrophil exhaustion relevant to sepsis pathogenesis.

Despite the well-recognized phenotype of neutrophil exhaustion, the underlying molecular mechanisms have not been well understood. Our data reveal that TICAM2 is contributing to the establishment of neutrophil exhaustion. In contrast to WT neutrophils exhausted by prolonged LPS challenge, TICAM2-deficient neutrophils challenged with LPS have reduced expression of adhesion molecules ICAM1, CD11a, CD11b, CD29 as well as immunosuppressive molecules PD-L1 and SIRPα. This correlates with reduced swarming of TICAM-2 KO neutrophils challenged with LPS. We also demonstrate that TICAM-2 KO neutrophils exhibit the attenuated aging phenotype as reflected on the relative retention of surface CXCR2 and CD62L molecules on TICAM-2 neutrophils following LPS challenge, as compared to the drastic reduction of CXCR2 and CD62L on WT neutrophils challenged with LPS. In addition to the in vitro observation, our current study provides evidence for the role of TICAM2 during neutrophil exhaustion in vivo. TICAM2 KO mice are protected from developing severe systemic inflammation and multi-organ damage triggered by DSS-induced mucosal injury. It is worth noting that innate exhaustion may not be limited to mature leukocytes such as neutrophils. Indeed, it was reported that LPS can drive the exhaustion of hematopoietic stem cell (HSC) with compromised expansion and renewal^68^ and that the TICAM2/TRIF signaling pathway contributes to the exhaustion of HSC^17^. Thus, it is likely that TICAM2 may also play a key role in HSC exhaustion affecting granulopoiesis, which requires more in-depth studies in the future.

Our data further reveal the significance of Src family kinase (SFK) during TICAM2 mediated neutrophil exhaustion. Extending upon the previous observation that SFK signals downstream of TLR4 in macrophages^69^, we demonstrated that LPS induces SFK activation with a particularly pronounced induction of Fyn kinase and STAT1 phosphorylation in neutrophils dependent upon TICAM2. Through the utilization of SFK inhibitor Dasatinib, we further provide a causal connection between SFK activation and neutrophil exhaustion. LPS-induced phosphorylation of STAT1 as well as the expression of ICAM-1 and PD-L1 are suppressed in the presence of Dasatinib, indicating that SFK is critical for the activation of STAT1, which regulates the expression of ICAM1 and PD-L1 associated with neutrophil exhaustion^70^. The application of Dasatinib also reduces the swarming of neutrophils challenged with LPS. Our data is consistent with a previous study demonstrating the role of STAT1 in sepsis pathogenesis. In an independent model of sepsis induced by cecal ligation and puncture, mice deficient in STAT1 were less susceptible to CLP-induced septic shock, indicating that STAT1 is critically involved in the development of systemic inflammation and the pathogenesis of sepsis^71^. However, we did notice that the application of SFK inhibitor only partially blocked neutrophil exhaustion phenotypes such as the surface expression of ICAM-1 and PD-L1. This suggests that the generation of neutrophil exhaustion is highly complex and likely involves additional signaling networks yet to be fully explored in future studies. Together, our data provide an important initial clue that TICAM-2 mediated STAT1 activation via SFK is at least partially involved in the establishment of neutrophil exhaustion and sepsis severity.

There are limitations associated with this study. The inhibitor approach may not be highly selective. SFK may be involved in additional biological processes of neutrophil activation in addition to the regulation of STAT1 and the expression of ICAM-1 and PD-L1^41,72,73^. We noticed a significant induction of Fyn by LPS, while the activation of Src is less pronounced. Although SFK is known to be induced in neutrophils by LPS, their roles in neutrophil exhaustion remain to be better clarified. Different family members of SFK may play distinct roles in the generation of unique aspects of neutrophil exhaustion, which needs to be better defined in future studies. Other signaling processes in addition to TICAM-2 mediated pathways may also be involved in neutrophil exhaustion. Given the emerging significance of neutrophil exhaustion in clinical medicine, future detailed studies are warranted to better define the complex networks that govern the dynamics of neutrophil activation/exhaustion at the single-cell level both in vitro and in vivo.

In conclusion, the present study reveals an important role of TICAM2 in the induction of neutrophil exhaustion. The deletion of TICAM2 can restore neutrophil homeostasis as well as protect the host from developing severe systemic inflammation and multi-organ injury. Our study suggests that TICAM2 may serve as a potential target in the future development of therapeutic strategies for the treatment of systemic inflammation and sepsis.

## Materials and methods

### Experimental animals

Studies were conducted following the guideline from NIH (National Institutes of Health), and approved by the Virginia Tech IACUC (Institutional Animal Care and Use Committee). TICAM2^−/−^ mice were obtained from Dr. Holger Eltzschig (University of Texas Houston).

### In vitro neutrophil culture

Neutrophils were harvested from bone marrow and isolated with 62.5% percoll gradient from C57BL/6 or TICAM2 KO mice as described previously^46^. Neutrophils were cultured as previously described with slight modification in complete RPMI medium containing 10% fetal bovine serum, 2 mM l-glutamine, and 1% penicillin/streptomycin, with 0.01 M HEPES as well as 100 ng/ml G-CSF. PBS or LPS (100 ng/ml) (Sigma, no. L2630)^46^.

### Flow analysis

Harvested cells were stained with antibodies including: anti-Ly6G (1:200 dilution; BioLegend, no. 127606, 127610, or 127618), anti-CD11b (1:200 dilution; BioLegend, no. 101206), anti-CD11a (1:200 dilution; BioLegend, no. 153103), anti-SIRPα (1:200 dilution; BioLegend, no. 37210), anti-CD62L (1:200 dilution; BioLegend, no. 104428), anti-PD-L1 (1:200 dilution; BioLegend, no. 124312), anti-CXCR2 (1:300 dilution; BioLegend, no. 149604), anti-ICAM1 (1:300 dilution; BioLegend, no. 116121), anti-CD29 antibodies (1:300 dilution; BioLegend, no. 102221). In some analysis mentioned in the results, cells were treated with a fixation kit (BD Biosciences), subsequently stained with anti-STAT1 (1:100 dilution; Cell Signaling, no. 80916S), anti-p-STAT1 (Tyr701) (1:100 dilution; Cell Signaling, no. 8009S), anti-p-Src (Tyr416) (1:20 dilution; ThermoFisher, no. MA5-28055), or anti-Fyn (1:50 dilution; Santa Cruz, no. sc-434 FITC). Surface phenotype, transcription factor and intracellular protein levels of Ly6G^+^ neutrophils were analyzed using FACSCanto II (BD Biosciences). Neutrophil viability was assessed by staining and flow analysis with the annexin V/PI kit (1:4,000 dilution; Thermo Fisher Scientific, no. P3566) as described previously^74^.

### Experimental DSS treatment

Multi-organ damage and systemic inflammation were induced in WT and TICAM2 KO mice (8–10 weeks, 25–30 g) by applying 4% DSS (MP Biomedicals) in drinking water for 6 days followed with 4 days of regular water. Mice conditions were closely monitored for the experimental periods as described^74^. After the end of the experimental regimen, tissues harvested from properly euthanized mice were used for ex vivo analysis as indicated in the results.

### LTB4 measurement

The LTB4 ELISA kit (R&D Systems) was used to measure the levels from the supernatant of cultured neutrophils following the instruction from the manufacture.

### Swarming assay

Neutrophils collected from the bone marrow stimulated with PBS or LPS (100 ng/ml) for 24 h were incubated in fibronectin-coated plates for 1 h. Polystyrene beads (size: 30 μm; Sigma, nos. 84135-5ML-F) were then added to the neutrophil culture in the ratio of 1 (bead):40 (cells) for 5, 15, 30, 45-min incubation. Images were taken at the end of each time point by the digital camera (AmScope, MU2003-BI) attached to the microscope. The size of swarming (the size of neutrophil aggregation attaching to the bead in each field) and the number of swarming events (beads that were attached by neutrophils per field) were calculated by NIH ImageJ software.

### In vitro SFK blockage

SFK inhibitor Dasatinib (100 nM) (Cayman Chemical, no. 11498) were applied to cultured neutrophils as indicted in the results for 24 h. Treated neutrophils were used for subsequent analysis as described in the results section.

### Histological analysis

Collected tissues were embedded in paraffin and sectioned into 5-μm slices as described^74^. Slides were stained with hematoxylin and eosin (H&E) or anti-MPO primary antibody (anti-MPO; dilution: 1:100; Abcam, no. ab9535) followed by a secondary antibody, ABC peroxidase (VECTOR, no. PK-6100), and DAB (VECTOR, no. SK-4100) as described previously with slight modification^74^. Twenty viewing fields from each sample were captured under the optical microscope. Pixel values reflecting the DAB stained color intensities of each viewing field were quantitated using the NIH ImageJ software.

### Statistical analysis

The software of Prism (GraphPad Software 8.0, La Jolla, CA) was used to conduct statistical analysis. The significance of the observations was evaluated by the statistical methods of Student’s t-test (among two groups), one-way ANOVA (among multiple groups), or two-way ANOVA (among multiple groups with different variables). p values were indicated on the corresponding figure legends.

## Supplementary information

Supplementary Information
