# Supplementary material for: TICAM2-related pathway mediates neutrophil exhaustion
Source: Sci Rep. 2020 Sep 1;10:14397. doi: 10.1038/s41598-020-71379-y (PMC7463027; doi:10.1038/s41598-020-71379-y)
Supplement: Supplementary file 1 — Supplementary Information [file 41598_2020_71379_MOESM1_ESM.docx]

***Supplementary Figures***

**TICAM2-related pathway mediates neutrophil exhaustion**

RuiCi Lin^1,2^, Yao Zhang^2^, Kisha Pradhan^2^, Liwu Li^1,2*^

^1^Translational Biology, Medicine, and Health Graduate Program; ^2^Department of Biological Sciences, Virginia Tech, Blacksburg, VA 24061


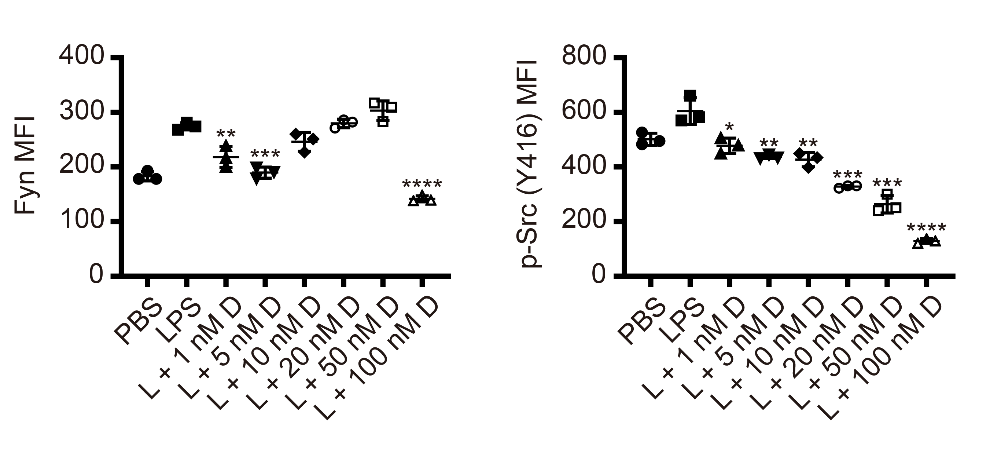


**Fig. S1** The dosage effect of Dasatinib on WT BM-derived neutrophils

Total Fyn and p-Src (Y416) expression on WT neutrophils treated with 0, 1, 5, 10, 20, 50, 100 nM Dasatinib in the presence of PBS or LPS (100 ng/ml) for 24 hours (n = 3).

All n-numbers represent data derived from individual cell cultures with data plotted as mean ± SD. ****P*<0.0001, ****P*<0.001, ***P*<0.01, **P*<0.05 using Student’s t-test, comparing with LPS group. L, LPS; D, Dasatinib.


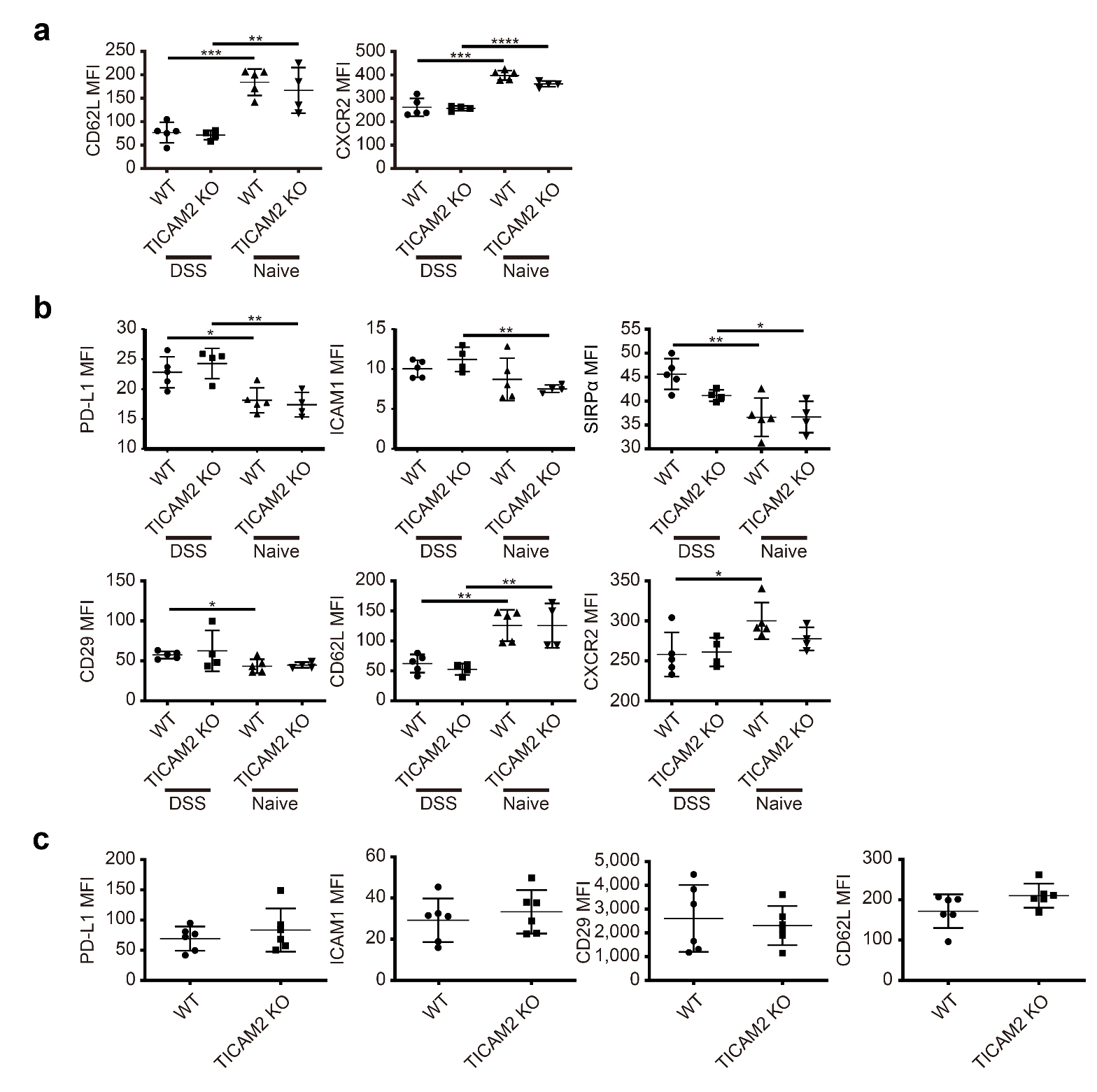


**Fig. S2** Neutrophils in blood and spleen from WT and TICAM2 KO, naïve and septic mice

**a** CD62L and CXCR2 expression on neutrophils in peripheral blood from mice with or without DSS treatment on day 6 (n = 4 or 5).

**b** The levels of PD-L1, ICAM1, SIRPα, CD29, CD62L, and CXCR2 on spleen-resident neutrophils from mice with or without DSS treatment on day 6 (n = 4 or 5).

**c** The expression of PD-L1, ICAM1, CD29, and CD62L on peripheral neutrophils from WT and TICAM2 KO septic mice on Day 10 (n = 5 or 6).

All n-numbers represent data derived from separate mice with data plotted as mean ± SD. *****P*<0.0001, ****P*<0.001, ***P*<0.01, **P*<0.05 (**a** and **b**). Data are representative of three independent experiments.
